# Selected papers from the 16th Annual Bio-Ontologies Special Interest Group Meeting

**DOI:** 10.1186/2041-1480-5-S1-I1

**Published:** 2014-06-03

**Authors:** Larisa N Soldatova, Philippe Rocca-Serra, Michel Dumontier, Nigam H Shah

**Affiliations:** 1 Brunel University, London, UK; 2 University of Oxford, Oxford e-Research Centre, UK; 3 Stanford University, CA, USA

## Abstract

Over the 16 years, the Bio-Ontologies SIG at ISMB has provided a forum for vibrant discussions of the latest and most innovative advances in the research area of bio-ontologies, its applications to biomedicine and more generally in the organisation, sharing and re-use of knowledge in biomedicine and the life sciences. The six papers selected for this supplement span a wide range of topics including: ontology-based data integration, ontology-based annotation of scientific literature, ontology and data model development, representation of scientific results and gene candidate prediction.

## Summary of selected papers

In 2013, the SIG received 26 submissions, including 15 papers, 5 flash updates and 6 poster abstracts. 7 papers and 6 flash updates (some papers were converted to flash updates) were selected for presentation at the meeting, out of which 6 appear in this supplement. The six papers selected for this supplement are extended versions of the original papers and flash updates presented at the 2013 SIG. The papers include research on such classic but nevertheless crucially important problems as ontology-based data integration [1-3], ontology-based annotation of scientific literature [1-4], ontology and data model development [2,3,5], representation of scientific results [5] and gene candidate prediction [6].

Bölling et al in the paper titled "SEE: structured representation of scientific evidence in the biomedical domain using Semantic Web techniques" present an RDF/OWL based approach for detailed representation of scientific evidences [1]. Knowledge in biomedicine is context-dependent and based on a variety of evidences obtained by experimental observations, inferences from other results, different interpretations, and modeling approaches. Bölling et al suggest RDO (the Reasoning and Discourse Ontology) - a lightweight OWL vocabulary for the representation and recording of how scientific claims are made and how they are related to each other. It provides computationally accessible representations of evidence-related information such as the materials, methods, assumptions and information sources used to establish a scientific finding. The proposed approach is demonstrated on the case study of evidence gathered in the literature regarding a claimed source of the enzyme glutamine synthetase. SEE resources, including the RDO ontology, are available from http://purl.org/see.

The paper titled "Statistical algorithms for ontology-based annotation of scientific literature" by Chakrabarti et al. reports on a probabilistic framework for annotating BrainMap literature using the Cognitive Paradigm Ontology (CogPO) [2]. This framework exploits hierarchical information, dependences and restrictions available in the ontology. At present, articles in the BrainMap repository are annotated manually according to CogPO definitions and it is a time and efforts intensive process that presents the major bottleneck for the whole repository. The proposed annotation framework would enable (semi-) automated solutions for the annotation of BrainMap literature. The proposed stochastic approaches for literature annotation were tested against the gold standard - the annotation by human subject matter experts, and yielded encouraging results.

Merrill et al in their paper "Semantic Web repositories for genomics data using the eXframe platform" addresses the critical task of the integration of genomic databases and data re-use [3]. They developed the second generation of the eXframe platform that supports the creation of online repositories to deposit genomics data as Linked Data. The eXframe platform provides a built-in SPARQL (Sparql Protocol and RDF Query Language) endpoint to query the data. The platform uses biomedical ontologies, e.g. OBI (the Ontology for Biomedical Investigations), DO (Disease Ontology), ChEBI (Chemical Entities of Biological Interests) ontology, to enable interoperability of the produced repositories. The platform also provides support for accessing data using popular statistical programming language R. The platform has been successfully tested through the case study of the Stem Cell Commons project of the Harvard Stem Cell Institute. eXframe is freely available at: https://github.com/mindinformatics/exframe.

Oellrich et al. in the paper titled "The influence of disease categories on gene candidate predictions from model organism phenotypes" analyse Exomiser's performance with respect to disease categories provided by Orphanet [4]. Exomiser is a tool previously developed by the authors to narrow down gene candidate lists that have been identified in exome analyses using cross-species phenotype comparisons amongst other sources of evidence. Oellrich et al. show that the prediction results depend on the organism and when automatically predicting disease gene candidates careful consideration is required as to which organism to apply for the predictions. For each disease category, they investigated the ten most common clinical phenotypes. Oellrich et al. found, for example, that the performance for zebrafish for nearly all disease categories is much more dependent on the disease category than it is for the mouse. The authors conclude that smarter tools capable of taking into account the differences between species and accumulate predictions are required.

The paper "Evolving BioAssay Ontology (BAO): modularization, integration and applications" by Abeyruwan et al. outline the work on the development of common reference metadata terms and definitions required for the reporting of information about low- and high- throughput drug and probe screening assays and results [5]. The authors have created BAO to support effective integration, aggregation, retrieval, and analyses of drug screening data. Abeyruwan et al. employed a modular approach for the development of BAO with domain-level components separated from structural components. The main components include bioassay, assay biology, assay method, assay format, assay endpoint and assay screened entity. BAO is sufficient to enable modeling of result profiles (signatures) generated in panel and profiling assays, for example those in the LINCS (the Library of Integrated Network-based Cellular Signatures) project. The authors have leveraged BAO in software tools, such as the Semantic Web software applications BAOSearch, LIFE, and the BioAssay Research Database (BARD). BAO is available at http://bioassayontology.org.

Tatum et al. in their paper titled "Preserving sequence annotations across reference sequences" present an RDF data model for describing sequence annotation instances within an established ontological framework that fits common practice of working with reference sequences and different versions of genome assemblies [6]. Tatum et al. created the Reference Sequence Ontology to provide a mechanism for linking annotation instances to different reference sequences. They also investigated how sequence annotations using different reference sequences can be semantically linked and identified three types of reference sequence relationships that are crucial for data integration. Tatum et al. present a working data model of sequence annotations that can be preserved across different reference sequence assemblies. The ontology of Reference Sequence Annotation is available at http://purl.bioontology.org/ontology/RSA.

## Competing interests

The authors declare that they have no competing interests.
